# Durability of CFRP–Steel Double–Lap Joints under Cyclic Freeze–Thaw/Wet–Dry Conditions

**DOI:** 10.3390/polym14173445

**Published:** 2022-08-24

**Authors:** Xiang Ren, Lingzhi Jiang, Jun He, Yi Yang, Yamin Sun, Qunfeng Liu, Shaojie Chen

**Affiliations:** 1 School of Architecture and Civil Engineering, Xi’an University of Science and Technology, Xi’an 710054, China; 2 School of Civil Engineering, Chang’an University, Xi’an 710061, China

**Keywords:** CFRP–steel joints, freeze–thaw/wet–dry environment, failure mode, ultimate load, degradation model

## Abstract

The usage of carbon fiber–reinforced polymer (CFRP) to strengthen cracked steel structures can effectively improve its bear capacity, so it has been extensively used in recent years. The degradation of interfacial bonding is one of the most important factors affecting the durability of CFRP–steel structures under a freeze–thaw(F–T)/wet–dry (W–D) environment. In this study, epoxy resin adhesive (ERA) dog-bone specimens and CFRP–steel double-lap joints (bonded joints) were prepared. F–T/W–D cycles experiment and tensile tests of the ERA specimens and the bonded joints were also performed. Under F–T/W–D cycles, the main properties of the ERA specimens and the bonded joints were examined. Results indicated that fracture failure occurred in all ERA specimens. The hybrid failure modes of fiber peeling on the surface of CFRP plate and the bonded interface peeling between the CFRP plate and ERA layer primarily occurred in the bonded joints. The failure of both of them can be considered to be brittle, which was unaffected by the F–T/W–D cycles. With increased F–T/W–D cycles, the ultimate load and tensile strength of the ERA specimens initially increased and then decreased, whereas the elastic modulus initially increased and then remained unchanged. The ultimate load of the bonded joints decreased gradually. Based on the relationship between the interfacial bond-slip parameters and the number of F–T/W–D cycles, the bond–slip model of the bonded joints was established. The proposed relationship was validated by comparing with the experimental bond-slip relationships and the predicted relationships under the F–T/W–D cycles.

## 1. Introduction

With the continuous increase in the total weight of operating vehicles and highway-traffic flow, the fatigue–cracking problem of existing steel bridges is becoming increasingly serious [1,2]. Drilling holes, welding, and additive steel-plate reinforcement methods are the traditional reinforcement or repair methods for cracked steel bridges. However, these methods have inherent defects, such as increased welding residual stress, local stress, and additional weight [3]. Carbon fiber-reinforced polymer (CFRP) has excellent properties such as light weight, high tensile strength, fatigue resistance, corrosion resistance, and convenience for construction. When it is attached onto the cracked part of the steel bridge, it can effectively improve the mechanical performance and does not excessively increase the dead weight of the bridge. Accordingly, the externally bonded CFRP–reinforcement technology has been used to rehabilitate steel bridges in recent years and has achieved good results [4,5,6,7]. The durability of rehabilitated structures by bonding CFRP plates depends on the bond performance of the reinforcement interface rather than the strength of the CFRP material itself. When the reinforced structures are exposed to the action of wet–dry(W–D), freeze-thaw(F–T) and other harsh environments for a long time, the performance of the bonded interface is reduced [8,9,10,11,12].

Epoxy resin adhesive (ERA) material is the main component of the bonded interface of CFRP–strengthened bridge structures, and its performance directly affects the bond properties of CFRP–steel double-lap joints [13,14]. The ERA is sensitive to ambient temperature and humidity [15], and it typically suffers from brittle failure, especially in cold environments [16,17]. Tensile strength also degrades with increased F–T cycle [18,19]. After reaching a certain cycle number, the tensile–shear strength returns to the unfrozen state [20]. The change in Young’s modulus is insignificant [21], and the elastic modulus of the ERA is hardly affected after 250 F–T cycles (−17 °C–8 °C) [22]. However, relevant studies have demonstrated that the tensile strength and elastic modulus of ERA increases after the first 50 F–T cycles owing to post-curing effect and continuously decreases after the second 50 F–T cycles [23]. The tensile-strength promotion of the ERA is primarily due to the release of internal stress caused by the diffusion of water molecules inside it [24].

Similarly, the performance of ERA also degrades when exposed to chlorine solution, seawater, and brine at different temperatures. The tensile strength and stiffness of the ERA specimens degrade at a high rate under seawater conditions of 20 °C and 50 °C [25], as well as brine at 40 °C for a long time [26]. Compared with fiber materials, the tensile properties of epoxy resin matrix are more sensitive to W–D exposure [27]. However, some studies have concluded that the influence on the performance of the ERA is minimal when exposed to chlorine solution for 36 months [28]. The salt solution accelerates the degradation of the ERA, and the presence of chlorine salt in F–T exposure aggravates the degradation [29].

CFRP–steel double-lap joints or single-lap joint specimens are often used to simulate steel bridges strengthened with CFRP. Borrie et al. [11] submerged CFRP–steel double-lap joints in a 5% NaCl solution for different durations up to 6 months at 20 °C, 40 °C and 50 °C, and subsequently conducted static tensile tests. Results show that overall, more than half of the specimens’ strength decreases by at least a 10%. In seawater at 20 °C and 50 °C, the degradation rate of strength and stiffness of the CFRP–steel specimens primarily occur in the initial stage, consistent with the degradation law of the ERA. This finding indicates that the degradation of the CFRP–steel specimens is primarily affected by the ERA [25]. In distilled water (20 °C, 45 °C) and 5% sodium chloride solution, the tensile strength and elastic modulus of the ERA decrease linearly with increased water content, but the ultimate load of FRP–steel specimen increase after soaking in distilled water and salt solution at 20 °C for one year [30]. In W–D cycle environment, the bear capacity of CFRP–steel beam decreases significantly and tends to decrease continually with increased W–D cycles [9]. Some studies have shown that the bearing capacity of CFRP–steel specimen tends to be stable after reaching a certain number of W–D cycles [31]. Bond-strength degradation is likely owing to the degradation of the bond property between the steel and the ERA layer. ERA-property degradation has also contributed to the property degradation of CFRP–steel specimens, but the role of this mechanism is apparently secondary [32,33].

Under F–T cycle condition (−16 °C–38 °C, 100% RH), the strength-reduction rate of CFRP–steel single-lap joints specimens in the early stage of F–T cycle is obviously higher than that in the late stage. The failure–mode change with increased F–T cycles [8]. The number of F–T cycles has no effect on the failure mode, the curve shapes of the interfacial shear stress distribution, and the bond–slip relationship of the steel-FRP lap joints. The bond–slip relationship curve is also approximately bilinear. However, the mechanical properties of the ERA and the steel–FRP lap joints specimens decrease with increased F–T cycles [34]. Conversely, other researchers have revealed that the tensile strength and elastic modulus of an ERA, Araldite–2015 (CN), and the ultimate load of the CFRP–steel specimen initially increase slightly and then decrease with increased F–T cycles (−20 °C–8 °C). The failure mode of all CFRP–steel specimens is mixed failure [23]. Yang et al. [35] studied the aging test of an ERA, SIKA30, and CFRP–steel joint in F–T environment (−20 °C–20 °C, 50% RH). Results showed that the tensile strength, elastic modulus, and ultimate strain of the ERA and the interfacial bond strength of the CFRP–steel specimen do not decrease after 10,000 h (883 cycles). However, the failure mode of the CFRP–steel specimen changes from cohesive failure to mixed failure after 10,000 h.

Heshmati et al. [36] soaked ERA and CFRP–steel specimens in 45 °C water or 45 °C saltwater for 36 months and then performed F–T experiments. Results show that the ERA ductility decreases after being dried from a wet state. However, such reduction is restored after the second absorption cycle. The failure mode of the CFRP–steel specimen pretreated with the salt solution changes, and the failure loads do not change significantly. This finding is due to the influence of water and temperature. The strength of CFRP–steel joints decreases after a complete W–D cycle in distilled water and saltwater, respectively. Their strengths are significantly larger than those observed after only wet exposure. Moreover, 125 and 250 F–T cycles are found to have no unfavorable effect on the strength of dry or preconditioned joints. Kim et al. [37] studied the bear capacity of FRP–steel joints under W–D and F–T cycles and found that the bond strength of components increases by 31.8% and 17.2%, respectively, after 100 cycles of W–D and F–T owing to the post-curing effect of water [33]. The interfacial stiffness of the specimen gradually decreases under the effects of W–D and F–T. The degradation of the specimen under the F–T cycle is more significant than that of W–D cycle. The failure mode of the specimen changes from cohesive failure to debonding failure.

In summary, ERA degradation in W–D and F–T environment is generally believed to be primarily caused by the hydrolysis of the ERA under the action of water molecules and the reversible softening reaction, thereby affecting the properties of the ERA [22,32,35]. For the bonded joints, first, the degradation of the bond strength and stiffness are controlled by bond-degradation properties of the bonded interface, although the deterioration rate of the strength and stiffness of the ERA and the bonded joints are basically the same. Second, the F–T, W–D cycles environment exert a dominant effect on the bond-property degradation of the bonded joints, and the effect is obviously greater on the ERA properties.

China has a vast territory and changeable climate. The south is wet and rainy, whereas the north is cold and dry. The cold region is extensively distributed, and the seasonal F–T environment is particularly obvious. Freeze and thaw environment in cold regions exist obviously with manmade chloride-corrosion environment by deicing method of salting snow on bridge deck in winter [38]. The action of salt corrosion and F–T environment leads to the infiltration of liquid into the interface of the bonded joints. The repeated action of salt corrosion, frost heaving, and melting aggravates the extension and propagation of microcracks in the bonded interface, which will seriously affect the service life of the bridge. Considering that the actual reinforced bridges continue to be affected by the effects of F–T/W–D, it is very necessary to study the long-term performance of CFRP steel double lap joints in the F–T/W–D environment.

The present study investigated the bond properties of the ERA and the bonded joints under the F–T/W–D interaction cycles through tests of the ERA and bond joints. The failure mode, ultimate load, and surface strain of the ERA and the bonded joints were tested. The main objectives of this study were as follow: (1) to study the effect of the F–T/W–D interaction cycle on the mechanical properties of the ERA and the bonded joints; (2) to analyze bond–slip relationship of the bonded joints under F–T/W–D interaction cycle; and (3) to simulate bond–slip degradation relationship of the bonded joints considering the F–T/W–D interaction cycle. The results can provide the basis for the development of the predictive degradation of ERA materials and the design of CFRP–strengthened structures.

## 2. Experimental Program

### 2.1. Material Properties

The steel plates used in the bonded joints were Q345B, whose characteristics were obtained by referring to the Chinese standard [39]. The ERA material was a mixture of two components A and B with a mass ratio of 2:1. The CFRP plate and ERA were produced by Nanjing Mankat Technology Co., Ltd., Nanjiang, Jiangsu Province, China, and their mechanical properties were provided by the manufacturer. Table 1 shows the material properties.

### 2.2. Specimens and Test Procedures

#### 2.2.1. ERA

**Figure 1:**
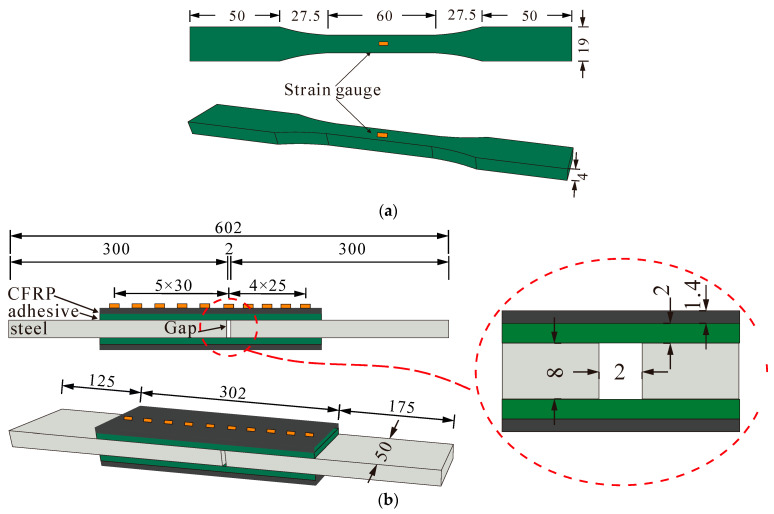
Geometry and strain gauge arrangement of the specimens. (Unit: mm). (**a**) The ERA specimen. (**b**) The CFRP–steel specimen.

A total of 15 specimens were prepared to investigate the effect of the F–T/W–D cycles on the mechanical properties of the ERA, as shown in Figure 1a. The ERA specimens were made according to GB/T2567–2008 [40]. According to the manufacturer’s recommendations, the ERA specimens were cured for at least 7 d at room temperature before testing. Three control specimens were tested directly after the ERA specimens were fully cured. Other ERA specimens were subjected to the F–T/W–D cycles after they were cured and subsequently tested according to ASTM D638–10 [41]. The ERA specimen identifications could be used the type of *Xi*-*m*, where *X* represents the F–T/W–D action, *i* is the number of F–T/W–D cycles, *m* is the number of specimen.

#### 2.2.2. Bonded Joints

A total of 15 specimens were made to study the effect of the F–T/W–D cycles on the bond properties of the bonded joints. The bonded-joints specimens were prepared according to ASTM D3528–96 (2008) and were tested according to ASTM D638–10 [40]. For the bonded joints, the length, width, and thickness of steel plates were 300, 50, and 8 mm, respectively. CFRP plates had identical length and width to those of steel plates. The thickness of the CFRP plates and ERA were 1.4 and 2.0 mm, respectively. The joint interval of steel plates was 2 mm, and the CFRP plate adopted asymmetric-bonding method. The bonding lengths on each side of the joints were 175 and 125 mm. The initial peeling of the bond interface was expected to occur in the shorter side direction, as shown in Figure 1b. The designation of the CFRP–steel joints was the same as that of the ERA specimens.

#### 2.2.3. Specimen Preparation

The interface-treatment method is one of the most important factors affecting the bond properties of bonded joints [42]. To improve the bond performance of the bonded joints, the steel plate surface was sandblasted [43], and the CFRP plate surface was evenly polished with 100 mesh fine sandpaper along the distribution direction of CFRP fiber before preparing the specimens. Then, the steel plate and CFRP plate surface were cleaned with acetone to ensure dryness and absence of dust. Afterwards, the CFRP plate was pasted on the both sides of the steel plate with 2 mm interval, and cured at room temperature in a specific mold for 7 d.

#### 2.2.4. Environmental Condition

In the F–T/W–D cycle experiment, F–T cycle was first conducted followed by W–D cycle. The environmental cycles were 0, 30, 60, 90, and 120 times. Cycle 0 represented no F–T/W–D cycle experienced and was defined as the control group. According to the slow F–T method in the Chinese National Standard GB/T-50082 [44], the temperature of the F–T cycle ranged from −20 °C to +30 °C, and the change from +30 °C to −20 °C (−20 °C to +30 °C) took 30 min at a rate of 1.2 °C/min. The time of the F–T cycle was around 8 h with 4 h of freezing and 4 h of thawing. The W–D environment was applied for 8 h per cycle while being soaked in 5% NaCl solution at room temperature for 4 h and air drying at room temperature for 4 h.

#### 2.2.5. Test Setup and Procedure

**Figure 2:**
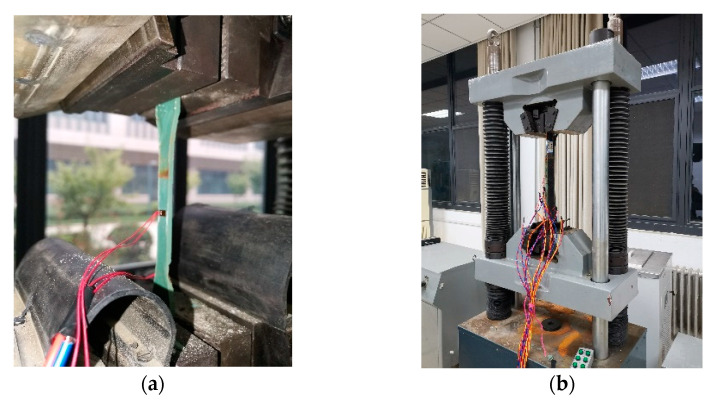
Schematic diagram of test setup. (**a**) The ERA specimen. (**b**) The bonded joints.

A WAW-300 microcomputer servo tester by Shenzhen Wance testing machine equipment Co., Ltd., Shenzhen, Guangzhou province, China, was adopted to conduct static tensile tests on ERA specimens and the bonded joints. Before loading, the upper and lower beams were adjusted, and the tensile force area of the specimen was completely exposed. Then, the upper and lower clips were adjusted to the clamp area of the specimen. The tensile–loading device is shown in Figure 2. The tensile test was loaded in a displacement-controlled manner to stretch the specimen at a tensile rate of 0.5 mm/min. To prevent the specimen from sliding between the upper and lower clips, the preload for 0.1–0.2 kN was performed. The failure mode, ultimate load, and surface strain of CFRP were recorded during tensile test.

## 3. Experimental Results

### 3.1. ERA Specimen

#### 3.1.1. Failure Modes

**Figure 3:**
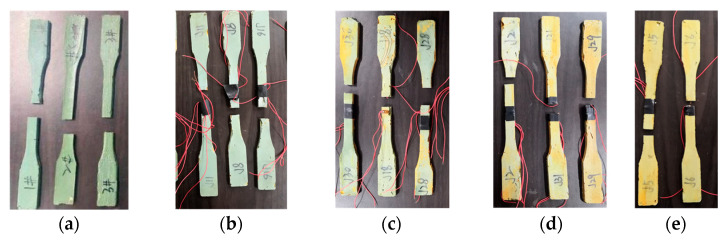
Failure mode of the ERA specimens. (**a**) 0 cycles, (**b**) 30 cycles, (**c**) 60 cycles, (**d**) 90 cycles, (**e**) 120 cycles.

The typical failure modes of the ERA specimen are shown in Figure 3. As shown in Figure 3, no obvious difference existed in the failure modes of all ERA specimens. The specimens had no neck shrinkage in the tensile process, and the fracture was sudden, indicating brittle characteristics.

#### 3.1.2. Ultimate Load and Peak Strain

The ultimate load and peak surface strain of the specimens are shown in Table 2. Table 2 shows that the ultimate load and surface strain of the ERA specimens initially increased and then decreased with increased F–T/W–D cycles. The largest values were in the 30 F–T/W–D cycles, which were around 44.7% and 147.1% higher than those of the control group, respectively. However, the smallest values were in the120 F–T/W–D cycles, which were around 18.1% and 1.1% lower than those of the control group, respectively. Analysis showed that a positive effect of the F–T/W–D cycles on the bearing capacity of the ERA occurred at the initial stage of the F–T/W–D cycles. However, the positive effect gradually weakened and became a negative impact with increased F–T/W–D cycles.

### 3.2. Bonded Joints

#### 3.2.1. Failure Modes

The bonded joints had six common failure modes (types A to F) [45]. Type A was CFRP delamination with fibers peeling of the CFRP surface, which may be due to the too thick ERA layer and the sufficient bond strength, which enabled easy peeling of the CFRP surface [46,47]. Type B was fracture of CFRP plate. In this failure mode, CFRP plate fractured transversely. Type C was the peeling failure of the bonded interface between the CFRP plate and ERA layer with no fiber adhering onto the surface of the ERA and no damage of the ERA layer. Type D was peeling failure of the bonded interface between the ERA layer and steel plate with no damage to the ERA layer. Type E was the damage of the ERA layer with ERA fractured or ERA debris adhered onto surface of the CFRP plate or the steel plate. Type F was steel-plate yielding with steel plate fractured or largely deformed.

**Figure 4:**
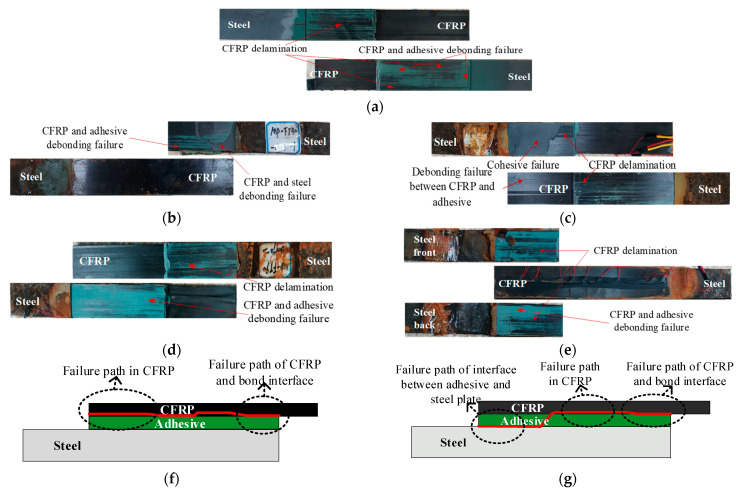
Typical failure modes of the specimens. (**a**) Type A + C (the control group). (**b**) A + C (the F–T/W–D30–1). (**c**) A + C + E (the F–T/W–D60–3). (**d**) A + C (the F–T/W–D90–3). (**e**) A + C (the F–T/W–D120–1). (**f**) Main failure mode Ⅰ of the bonded joints. (**g**) Main failure mode Ⅱ of the bonded joints.

The typical failure modes of the bonded joints are shown in Figure 4. Experimental results showed that the F–W/W–D cycles had little effect on the failure mode, and most specimens underwent type A + C failure. The exceptions were specimens FT30–DW30–1, FT30–DW30–2, and FT60–DW60–3, which underwent type A + C + E failure as shown in Table 3. Furthermore, most specimens underwent bonded-interface failure in the shorter side of the CFRP plate. Therefore, the experimentally observed diagonal failure could be explained as it provided the weakest path to failure. This finding was due the fact that once interface cracks appeared, the axis of the specimen deviated laterally and was no longer in axis tension [48]. The strength of the bonded joints primarily depended on the fiber interlayer strength and the bond strength of the interface between the CFRP plate and ERA [32]. The strength of the ERA played a secondary role for the ultimate bearing capacity of the bonded joints. However, the strength of the steel plate had no effect on the strength of the bonded joints [31,32]. The bond-interface failure characteristics that the carbon fiber attached onto the surfaces of the ERA layer gradually decreased with increased F–T/W–D cycles, and the area of type C increased.

#### 3.2.2. Ultimate Load and Peak Strain

The ultimate load of the bonded joints and the peak surface strain of CFRP plate were recorded during the tensile process of all specimens, as shown in Table 3. Compared with the control group, the ultimate loads of the F–T/W–D specimens gradually decreased. Under the 120 F–T/W–D cycles, the ultimate load decreased by 58.0%. Further comparison showed the influence of the F–T/W–D cycles on the ultimate load of the ERA specimens and the bonded joints were consistent.

## 4. Discussion

### 4.1. ERA Specimen

#### 4.1.1. Stress–Strain Relationship Curve

**Figure 5:**
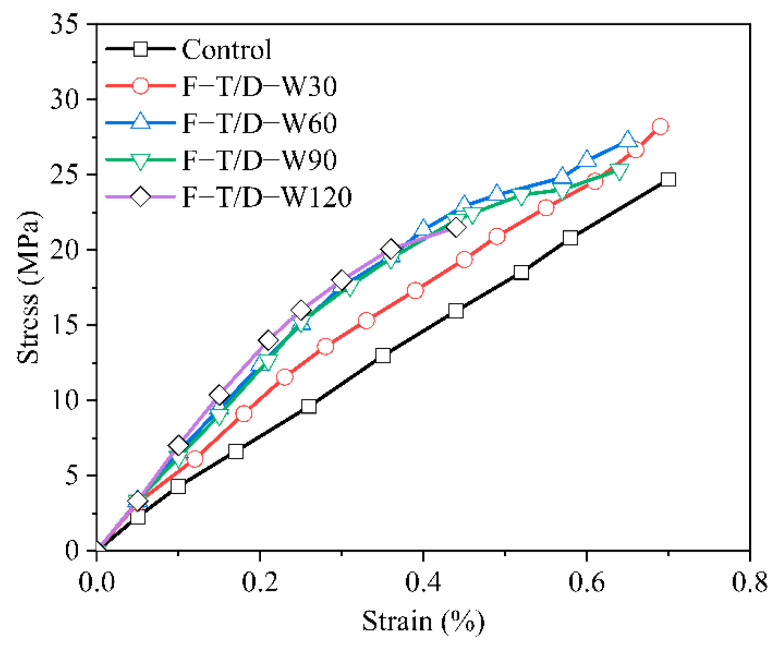
The stress–strain relationship curves of the ERA specimens.

Figure 5 shows the stress–strain curve of the ERA under each F–T/W–D cycles. The stress–strain relationship curve of the control group showed significant linearity. With increased F–T/W–D cycles, the stress–strain curve of the ERA specimens presented certain nonlinearity, which became particularly obvious after the 60 F–T/W–D cycles.

#### 4.1.2. Strength and Elastic Modulus of the ERA

**Figure 6:**
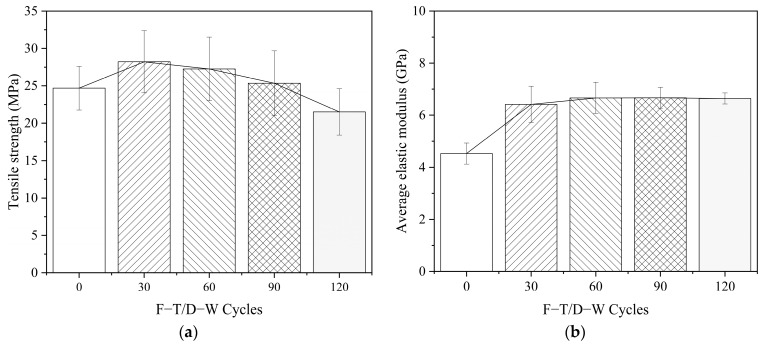
Mechanical property of the ERA. (**a**) Tensile strength, (**b**) Elastic modulus.

Tensile strength is expressed by the ratio of the ultimate load to the minimum cross–section area of the ERA specimens. The elastic modulus is taken as the maximum slope of the stress–strain relationship curve [49]. From 0, 30, 60, 90, to 120 F–T/W–D cycles, the tensile strength of the F–T/W–D specimens initially increased and then decreased, as shown in the Figure 6a. The elastic modulus initially increased and then unchanged basically, as shown in Figure 6b. This result indicated that the post–curing of ERA played a dominant in the initial stage of F–T/W–D exposure. And as the post–curing was gradually completed, the negative temperature in the F–T environment interrupted the curing process, resulting in a nearly constant elastic modulus [50,51]. Compared with the control specimen, the tensile strength increased around 14.3% under the 30 F–T/W–D cycles and decreased by around 12.9% under the 120 F–T/W–D cycles. The elastic modulus increased by 41.7% and 46.7% under 30 and 120 F–T/D-W cycles, respectively.

The F–T/W–D specimens underwent a certain number of F–T cycles, and the water penetrated the interior of the ERA layer. Consequently, a curing reaction occurred and the tensile strength of the ERA specimens improved. With further freezing action, cracks occured in the interior of the ERA specimens owing to water expansion. Chloride ions in the sodium chloride solution entered the ERA–layer interior through cracks and underwent hydrolysis under the action of the W–D cycle, resulting in a sudden drop in tensile strength of the ERA [36]. Analysis results showed that the presence of salt solution in the F–T environment accelerated the degradation of the ERA’s mechanical properties.

### 4.2. Bonded Joints

#### 4.2.1. Surface Strain Distribution

By collecting the surface strain of the CFRP plate under different loaded stages, the relationship curve between the axial strain distribution of the CFRP plate surface and different loads was obtained. Then the adhesive failure process of the specimens during the tensile process was analyzed.

**Figure 7:**
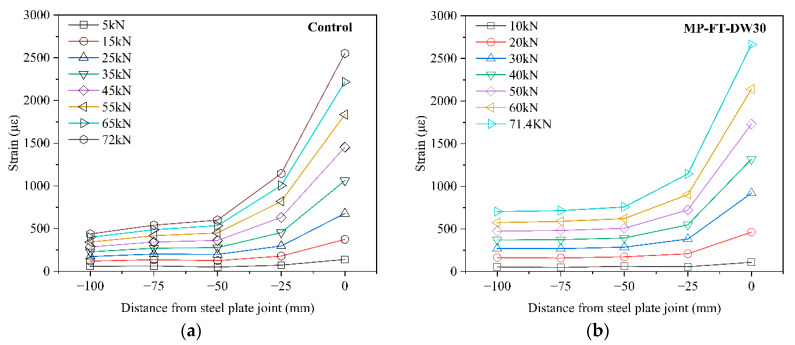

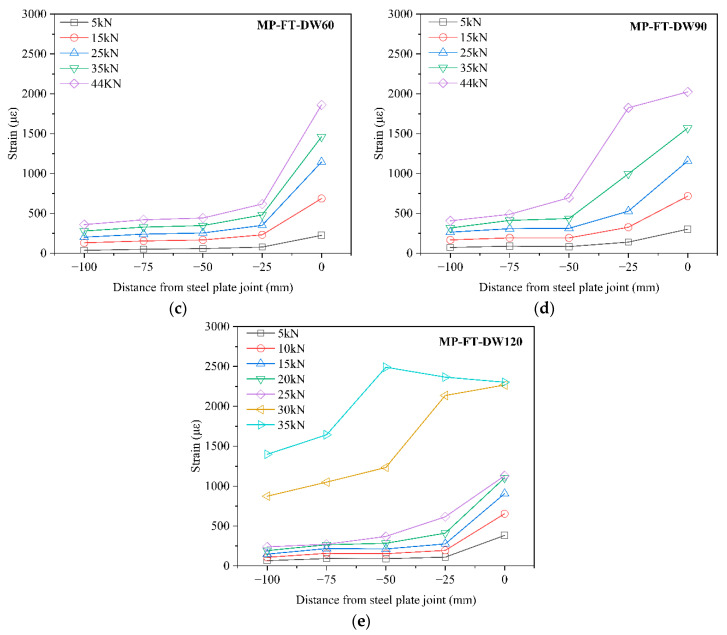
Strain distributions of the CFRP plate surfaces. (**a**) control group. (**b**) 30 F–T/W–D cycles. (**c**) 60 F–T/W–D cycles. (**d**) 90 F–T/W–D cycles. (**e**) 120 F–T/W–D cycles.

Figure 7 shows the axial strain distributions along the CFRP plate surface. As shown in Figure 7, for the control group and the F–W/W–D specimens, the strain distributions on the CFRP plate surface were basically similar. The peak strains of the CFRP plate always appeared at the interval of the two steel plates (a strain gauge was attached near here). Further analysis of the strain distribution on the CFRP plate surface showed that the strain decreased sharply from the peak strain point to the adjacent measuring points. Then, the strain changed little toward the load end, and the strain value was close to zero.

The strain concentration area of the bond interface was located near the interval of the two steel plates, and the concentration area was small. With increased F–T/W–D cycles, when the specimen approached the ultimate load, the CFRP surface strain near the interval reached the peak value, and then the value slightly decreased with increased F–T/W–D cycles, particularly after 60 F–T/W–D cycles.

#### 4.2.2. Bond-Slip Relationship

Bond-slip relationship refers to the relationship between the local shear stress and the corresponding slip along the bonded interface of the bonded joints [50]. The bond–slip relationship can be used as the constitutive relationship of the bond interface and could predict the interface failure process of the steel bridge strengthened with CFRP plate. It can characterize the local stress and bond-failure process of the bond interface [51].

Interfacial shear stress and slip can be observed by reading the strain attached onto the surfaces of the CFRP plate by using Equations (1) and (2) [50,52]:

(1) $$\tau_{i-1/2}=\frac{\varepsilon_{i}-\varepsilon_{i-1}}{L_{i}-L_{i-1}}E_{p}t_{p}$$

(2) $$S_{i-\frac{1}{2}}=\frac{\left(\varepsilon_{i}+\varepsilon_{i-1}\right)}{4}\left(L_{i}-L_{i-1}\right)+\frac{\left(\varepsilon_{i-1}+\varepsilon_{i-2}\right)}{2}\left(L_{i-1}-L_{i-2}\right)+\sum_{i=3}^{i}\frac{\left(\varepsilon_{i-2}+\varepsilon_{i-3}\right)}{2}\left(L_{i-2}-L_{i-3}\right)$$

where *ε_i_* is the reading of the *i*th strain gauge counted from the free end of CFRP plate, *ε_0_* = 0; *L_i_* is the distance of the *i*th strain gauge from the free end of the CFRP plate, *L*_0_ = 0; *E_p_* and *t_p_* are the elastic modulus and thickness of the CFRP plate, respectively; and *τ_i_*_−1/2_ and *S_i_*_−1/2_ are the shear stress and slip at the middle point between the *i*th strain gauge and the *i*–*i*th strain gauge.

**Figure 8:**
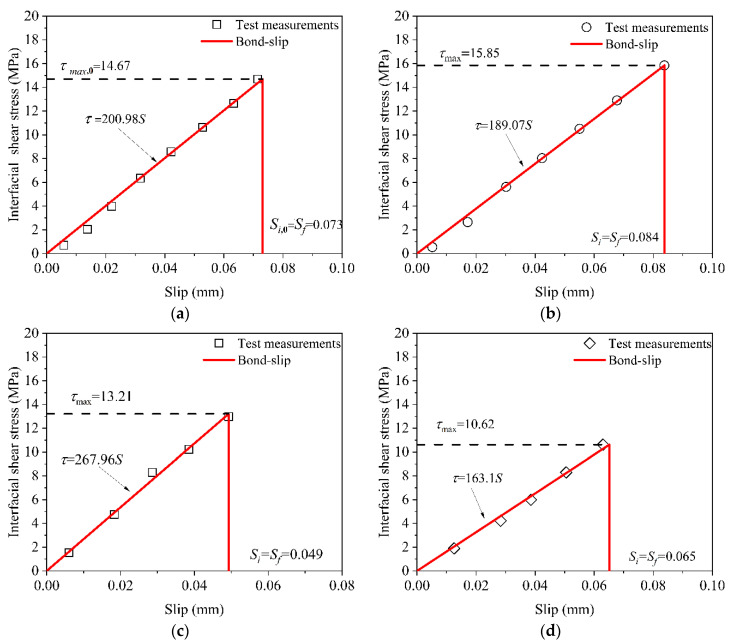

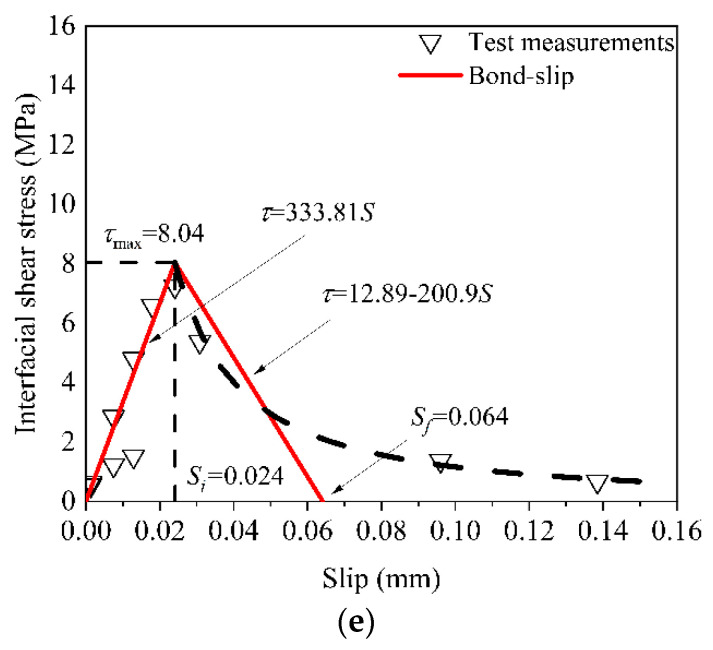
The bond-slip curves of the bonded joints. (**a**) control group. (**b**) 30 F–T/W–D cycles. (**c**) 60 F–T/W–D cycles. (**d**) 90 F–T/W–D cycles. (**e**) 120 F–T/W–D cycles.

As shown in Figure 8, the bond–slip curves were obtained by adopting Equations (1) and (2). As shown in Figure 8, the bond-slip curve under the 120 F–T/W–D cycles had an ascending branch and a descending branch of the bonded joints. However, the bond–slip curves of the control specimens and environmental specimens under the 30, 60, and 90 F–T/W–D cycles had no descending branch. The bond–slip curves of all specimens had a similar bi–linear shape [50,53].

The bond-slip relationship of all specimens can be expressed approximately as Equation (3) [23,50].

(3) $$\left\{ \begin{array}{l} \tau =\tau_{\max}\frac{S}{S_{1}},S\leq S_{1}\\ \tau =\tau_{\max}\frac{S_{f}-S}{S_{f}-S_{1}},S_{1}<S\leq S_{f}\\ \tau =0,S>S_{f} \end{array}\right.$$

where τ is the interfacial shear stress; $\tau_{\max}$ is the maximum interfacial shear stress; S is the slip value corresponding to the shear stress τ; $S_{1}$ is the slip value corresponding to the maximum interfacial shear stress $\tau_{\max}$; and $S_{f}$ is maximum slip value. The specific expression is shown in Figure 8.

## 5. Interfacial Bond-Slip Degradation Relationship

### 5.1. Interfacial Bond-Slip Parameters

The parameters reflecting the interfacial bond properties of the bonded joints are *τ*_max_, *S*_1_, *S_f_*, and *G_f_*. The parameters *τ*_max_, *S*_1_, and *S_f_* can be obtained from the bond-slip curve in Figure 8. The parameter *G_f_* is approximately expressed as the envelop area of the bond–slip curve [23]. All parameters are listed in Table 4.

### 5.2. Degradation Models of Interfacial Bond-Slip Parameters

The damage factor D(n) was introduced to describe the degradation of the interfacial bond-slip parameters after the F–T/W–D cycles. The damage factor D(n) was determined by Equation (4) [23]:

(4) $$D(n)=1-X(n)/X_{0}$$

where X(n) is the bond-slip parameters after *n* F–T/W–D cycles, and $X_{0}$ is the parameters value without F–T/W–D cycles.

**Figure 9:**
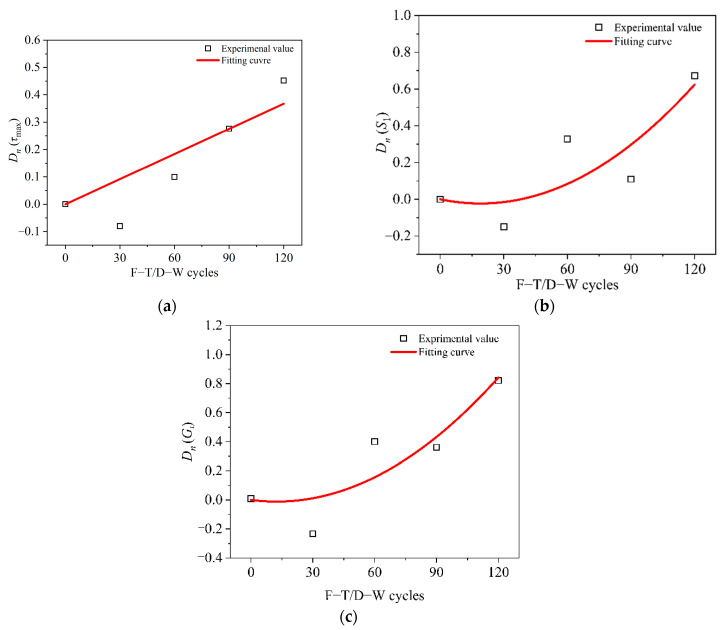
Degradation evolution of the interfacial bond-slip parameters. (**a**) Maximum shear stress *τ*_max_, (**b**) Relative slip *S*_1_. (**c**) Interfacial fracture energy *G_f_*.

The values of the parameter *S*_1_ were the same as those of the parameter *S_f_* for the specimens of 30, 60, and 90 F–T/W–D cycles. However, for the specimen of 120 F–T/W–D cycles, the experience time from *S*_1_ to *S_f_* was very short, so the parameter *S_f_* was not discussed in this paper. Figure 9 presentes the damage evolution of the interfacial bond–slip parameters after F–T/W–D cycles. Compared with the control specimen, the damage factors of the parameter *D*(*τ*_max_), *D*(*S*_1_) and *D*(*G_f_*) slightly decreased under the 30 F–T/W–D cycles because of the interfacial enhancement owing to the post-curing of the adhesive [23]. However, they increased after 60 F–T/W–D cycles. Equations (5) and (6) were used to fit the variation trend of the parameters with increased F–T/W–D cycles.

(5) D(n)=an

(6) $$D\left(n\right)=bn^{2}+cn$$

where *n* is the number of the F–T/W–D cycle; and *a*, *b* and *c* are the fitting coefficients of Equations (5) and (6) as shown in Table 5. The expressions of the interfacial bond-slip parameters were obtained by substituting Equations (5) and (6) into Equation (4):

(7) $$X(n)=X_{0}\left(1-D(n)\right)=\{{}_{X_{0}\left[1-(bn^{2}+cn)\right]}^{X_{0}\left(1-an\right)}\begin{array}{l} \;X=\tau_{\max}\\ \;X=S_{1},G_{f} \end{array}$$

Formula (7) can express the relationship between the parameter *τ*_max_, *S*_1_, and *G_f_* of the bonded joints and the number of F–T/W–D cycles.

### 5.3. Bond-Slip Degradation Relationship

**Figure 10:**
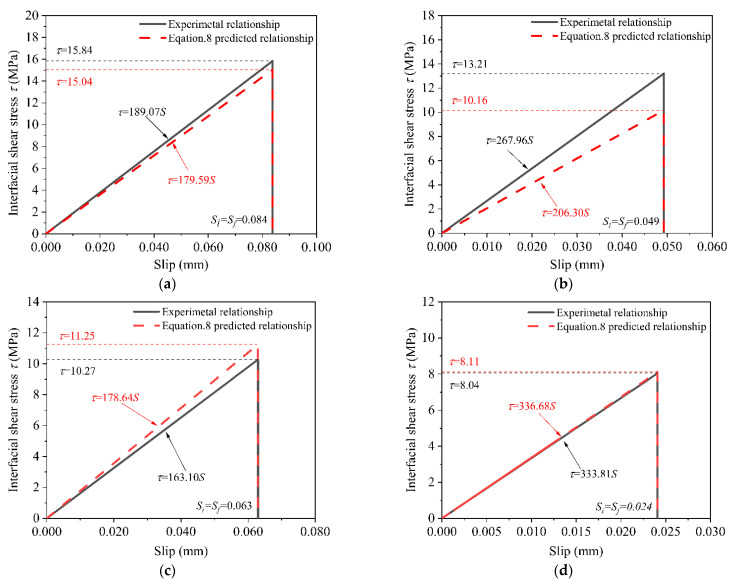
Comparisons of the experimental and predicted bond-slip degradation curves. (**a**) 30 F–T/W–D cycles. (**b**) 60 F–T/W–D cycles. (**c**) 90 F–T/W–D cycles. (**d**) 120 F–T/W–D cycles.

The interfacial bond-slip degradation relationship was obtained by substituting Equation (7) into Equation (2), yielding Equation (8):

(8) $$\left\{ \begin{array}{l} \tau =\frac{\tau_{\max,0}\left(1-an\right)}{S_{0}\left[1-\left(bn^{2}+cn\right)\right]}S,S\leq S_{0}\left[1-\left(bn^{2}+cn\right)\right]\\ \tau =0,S>S_{0}\left[1-\left(bn^{2}+cn\right)\right] \end{array}\right.$$

where $\tau_{\max,0}$ and $S_{0}$ are the maximum interfacial shear stress and corresponding slip value without F–T/W–D cycles, respectively. Figure 10 shows the comparisons of the predicted and experimental bond–slip degradation relationship. The predicted degeneration relationship well agreed with experimental ones, except for specimens under the 60 F–T/W–D cycle, which had 23.1% maximum deviation between the maximum experimental shear stress value and the maximum predicted.

## 6. Conclusions

Based on the experimental results and analysis of this research, the following conclusions were drawn.

(1) Fracture failure occurred within the area of a small section for all ERA specimens. The hybrid failure modes of fiber peeling on the surface of CFRP plate and bonded interface peeling between CFRP plate and ERA layer primarily occurred in the bonded joints. A few ones had ERA layer failure, and the fracture failure both of the ERA specimens and the bonded joints can be considered to be brittle, indicating that they were unaffected by the F–T/W–D cycles.

(2) With increased F–T/W–D cycles, the ultimate load and tensile strength of the ERA specimens initially increased and then decreased, whereas the elastic modulus basically remained unchanged. For the bonded joints, the ultimate load decreased gradually, and the peak strain of CFRP plate surface appeared at the interval of steel plates, before sharply decreasing toward the loading end.

(3) The triangular double-line model was used to describe the interfacial bond-slip relationship of the bonded joints under the F–T/W–D cycles. The expression of the bond-slip relationship was obtained, and the degradation models of the maximum peak shear stress and the corresponding slip value with F–T/W–D cycles were established based on the fitting. Then, the bond-slip relationship models of the bonded joints were proposed, and the models were verified by comparing with the experimental ones. On this basis, the bond-ship relationship under F–T/W–D cycles was predicted.

## Figures and Tables

**Table 1:** The mechanical properties of the materials.

Material Type	Tensile Strength (MPa)	Tensile Modulus (GPa)	Elongation Break (%)	Shear Strength (MPa)
CFRP	2400	160	1.65	50.5
ERA	30.3	4.92	1.62	/
Steel	455	206	/	/

**Table 2:** Ultimate load and Peak strain of the ERA.

Specimen Number	Ultimate Load (kN)	Mean Value (kN)/SD	Peak Strain (με)	Mean Value (με)/SD
C0–1	0.94	0.94/±0.04	3433	3930/±418
C0–2	0.99		4457	
C0–2	0.90		3899	
FT/DW30–1	1.29	1.36/±0.05	9629	9712/±84
FT/DW30–2	1.37		9827	
FT/DW30–3	1.42		9680	
FT/DW60–1	1.31	1.32/±0.07	4064	4880/±1498
FT/DW60–2	1.41		6982	
FT/DW60–3	1.24		3596	
FT/DW90–1	1.19	1.23/±0.04	5310	5344/±48
FT/DW90–2	1.22		5412	
FT/DW90–3	1.28		5311	
FT/DW120–1	0.66	0.77/±0.12	4082	3885/±377
FT/DW120–2	0.93		4215	
FT/DW120–3	0.72		3357	

**Table 3:** Ultimate load, interfacial peak strain and main failure modes of the bonded joints.

Specimen No.	Ultimate Load (kN)	Mean Value (kN)/SD	Peak Strain (με)	Mean Value (με)/SD	Failure Mode
C–0–1	75.0	74.0/±1.4	2252	2338/±86	A + C
C–0–2	73.0		2424		A + C
FT30–DW30–1	68.8	67.0/±4.5	2769	2487/±327	A + C + E
FT30–DW30–2	60.9		2028		A + C + E
FT30–DW30–3	71.4		2664		A + C
FT60–DW60–1	40.0	45.0/±4.5	1614	1741/±101	A + C
FT60–DW60–2	44.2		1862		A + C
FT60–DW60–3	50.9		1746		A + C + E
FT90–DW90–1	44.1	45.4/±1.4	2025	2286/±261	A + C
FT90–DW90–2	30.2		366		A + C
FT90–DW90–3	46.7		2546		A + C
FT120–DW120–1	28.2	31.1/±3.0	1894	2119/±169	A + C
FT120–DW120–2	48.3		2161		A + C
FT120–DW120–3	34.1		2302		A + C

A = CFRP delamination; C = Interfacial failure between the CFRP and ERA layer; E = ERA fractured (ERA debris adhered onto surface of the CFRP plate or the steel plate). The acronyms A + C, A + C + E are represented by a mixed damage mode.

**Table 4:** Bond performance parameters of the CFRP–steel specimen.

Specimen Number	$\tau_{\max}\;(\mathrm{MPa})$	$S_{1}\;(\mathrm{mm})$	$S_{f}\;(\mathrm{mm})$	$G_{f}\;(N/\mathrm{mm})$	Average Value of Ultimate Load (kN)
MP–FT–DW–0	14.67	0.073	0.073	0.54	74.0
MP–FT–DW–30	15.85	0.084	0.084	0.67	67.0
MP–FT–DW–60	13.21	0.049	0.049	0.32	45.0
MP–FT–DW–90	10.62	0.065	0.065	0.35	45.4
MP–FT–DW–120	8.04	0.024	0.064	0.10	31.1

**Table 5:** The fitting coefficients *a*, *b* and *c*.

Bond-Slip Parameter	*a*	*b*	*c*
$\tau_{\max}$	0.0031	/	/
$S_{1}$	/	6.3414 × 10^−5^	−0.0024
$G_{f}$	/	7.3647 × 10^−5^	−0.0018

## Data Availability

The data presented in this study are available on request from the corresponding author.
